# The effect of salinity on *enterocytozoon hepatopenaei* infection in *Penaeus vannamei* under experimental conditions

**DOI:** 10.1186/s12917-021-02778-0

**Published:** 2021-02-02

**Authors:** L. F. Aranguren Caro, F. Alghamdi, K. De Belder, J. Lin, H. N. Mai, J. Millabas, Y. Alrehaili, A. Alazwari, S. Algetham, A. K. Dhar

**Affiliations:** 1 Aquaculture Pathology Laboratory, School of Animal and Comparative Biomedical Sciences, The University of Arizona, 1117 E Lowell st. Tucson, AZ,, 85721 USA; 2 Health & Fisheries Services Ministry of Environment, Water and Agriculture, 65 King Abdulaziz Road, Riyadh, 11195 Kingdom of Saudi Arabia

**Keywords:** *Enterocytozoon hepatopenaei*, EHP, Salinity, *Penaeus vannamei*

## Abstract

**Background:**

*Enterocytozoon hepatopenaei* (EHP) is an enteric pathogen that affects *Penaeus vannamei* and *Penaeus monodon* shrimp in many SE Asian countries. In the western hemisphere, EHP was reported for the first time in 2016 in farmed *P. vannamei* in Venezuela. Anecdotal evidence suggests that EHP is more prevalent in grow-out ponds where the salinity is high (> 15 parts per thousand (ppt)) compared to grow-out ponds with low salinities (< 5 ppt). Considering that *P. vannamei* is an euryhaline species, we were interested in knowing if EHP can propagate in *P. vannamei* in low salinities.

**Results:**

In this study, we described an experimental infection using fecal strings as a source inoculum. Specific Pathogen Free (SPF) *P. vannamei* were maintained at three different salinities (2 ppt, 15 ppt, and 30 ppt) while continuously challenged using feces from known EHP-infected *P. vannamei* over a period of 3 weeks. The fecal strings, used as a source of EHP inocula in the challenges, was sufficient to elicit an infection in shrimp maintained at the three salinities. The infectivity of EHP in shrimp reared at 2 ppt, 15 ppt, and 30 ppt salinities was confirmed by PCR and histopathology. The prevalence and the severity of the EHP infection was higher at 30 ppt than at 2 ppt and 15 ppt.

**Conclusion:**

The data suggests that fecal strings are a reliable source of EHP inoculum to conduct experimental challenges via the fecal-oral route. An EHP infection can occur at a salinity as low as 2 ppt, however, the prevalence and the severity of the EHP infection is higher at a salinity of 30 ppt.

## Background

Hepatopancreatic microsporidiosis (HPM) caused by *Enterocytozoon hepatopenaei* (EHP) is a disease that has been reported in several penaeid shrimp including *P. monodon* and *P. vannamei* [1, 2]. EHP has been reported in different regions including Asian countries such as China, Indonesia, Malaysia, Vietnam, Thailand, and India [3–6]. Recently, EHP has also been reported in Venezuela located in the western hemisphere [7].

The main clinical signs of EHP are growth retardation [2, 8] that leads to increased size variability. Whitish fecal strings floating on the surface of pond water and the presence of shrimp displaying white discoloration of the gastrointestinal tract (GI tract) in these ponds have also been associated with EHP [3, 9]. In advanced stages of the disease, EHP-infected shrimp typically display soft shells, lethargy, reduced feed intake, an empty midgut, and chronic mortalities. EHP is an intracellular microsporidium that causes lesions in the hepatopancreas (HP) tubule epithelial cells. EHP replicates within the cytoplasm of the affected cells. Histology of EHP-infected shrimp shows irregular/regular basophilic inclusion bodies within the cytoplasm with or without the presence of spores. Additional histological lesions include mild to severe sloughing of the tubular epithelial cells usually with presence of mature spores. Moreover, the spores are also observed in the lumen of the HP tubules and the GI tract [2, 9].

In countries where EHP has been reported, such as India, China, Vietnam, and Venezuela, shrimp farming is carried out in a wide range of environmental condition including coastal marine areas, estuarine areas, and inland areas. For instance, in some states in eastern part of India such as Andhra Pradesh, the ponds are filled with borehole water mixed with estuarine water, which causes the salinities of the grow-out ponds to fluctuate between 0 and 30 ppt with an average salinity of about 10 ppt. In contrast, in states located in the western part of the country such as Gujarat, salinities in pond water may vary between 30 and 44 ppt [10, 11]. In Venezuela on the western hemisphere, the shrimp farms are located in two areas. One of these areas is in the Maracaibo lake where the salinity ranges between 2 and 5 ppt. The other area is located in the northern part of the country where shrimp farms are located in the marine environment where salinity ranges between 20 and 40 ppt [12] . Interestingly, in both environments of high and low salinity, EHP has been reported. In these countries, the incidence of EHP seems to be higher in high salinity environments, but there is no study to support the possible relationship between salinity and the presence of EHP. For this reason, the objective of this study was to compare the infectivity of EHP using fecal strings as inoculum in three different salinities under experimental conditions.

## Results

### Survival rates

The final survival at the end of the EHP challenge was high, ranging from 90 to 100%, in the three salinities in each of the two independent experimental challenges (Table 1). The survival percentage in the control treatments were 100% in all of the salinities in the two independent challenge experiments. We did not observe any clinical signs in the shrimp exposed to EHP in the 3 different salinities.

**Table 1:** Final survival in *Penaeus vannamei* challenged with fecal string as EHP inoculum in two independent experimental challenges at three different salinities. The data represented as Mean ± SD

Experimental Challenge	Duration	Final survival (%)					
		2 ppt EHP treatment	2 ppt control	15 ppt EHP treatment	15 ppt control	30 ppt EHP treatment	30 ppt control
1	20 days	95 ± 7	100 ± 0	100 ± 0	100 ± 0	100 ± 0	100 ± 0
2	26 days	90 ± 0	100 ± 0	100 ± 0	100 ± 0	90 ± 7	100 ± 0

### Quantification of EHP in fecal strings by qPCR

Fecal strings were collected on a daily basis from known EHP-infected tanks. The daily fecal string samples tested positive for EHP in both challenges. The average weight of the fecal strings added to each tank was 1.17 ± 0.52 g and 0.32 ± 0.24 g for challenges #1 and #2, respectively.

**Fig. 1:**
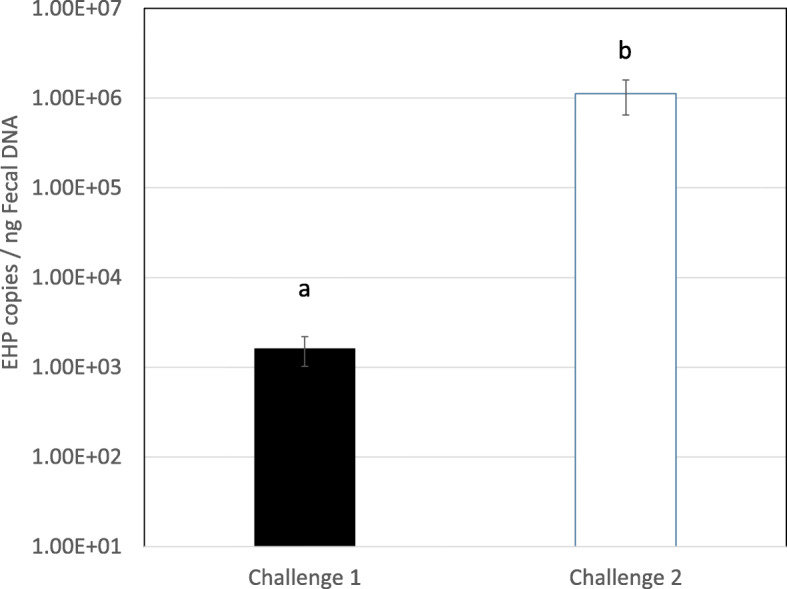
EHPcopynumberinfecalstringsaddeddailytothetanksinchallenge#1and#2.SamplesanalyzedbyReal-timePCR.DatasetisrepresentedwiththeMean±SE.Lettersaandbindicatesignificantdifferences (*p* < 0.05)

The EHP copy number in the fecal strings used as inoculum was significantly higher in the challenge #2 (*p* < 0.05). EHP copy number in the fecal string in challenge #1 was 1.6 × 10^3^ ± 2.1 × 10^3^ copies /ng DNA compared to 1.1 × 10^6^ ± 2.0 × 10^6^ copies /ng DNA in the inoculum of challenge #2 (Fig. 1).

### Prevalence of EHP by histopathology

The prevalence and severity of EHP in shrimp experimentally challenged using fecal strings as inocula in the two independent challenges was assessed by H&E histology.

In both experimental challenges, the fecal strings used as inocula were able to provoke the disease in SPF shrimp. The prevalence of EHP was 28.5% in the challenge #1 vs. 50.0% in the challenge #2. The data confirms that fecal strings are a reliable source of inoculum for experimental infection of EHP.

**Fig. 2:**
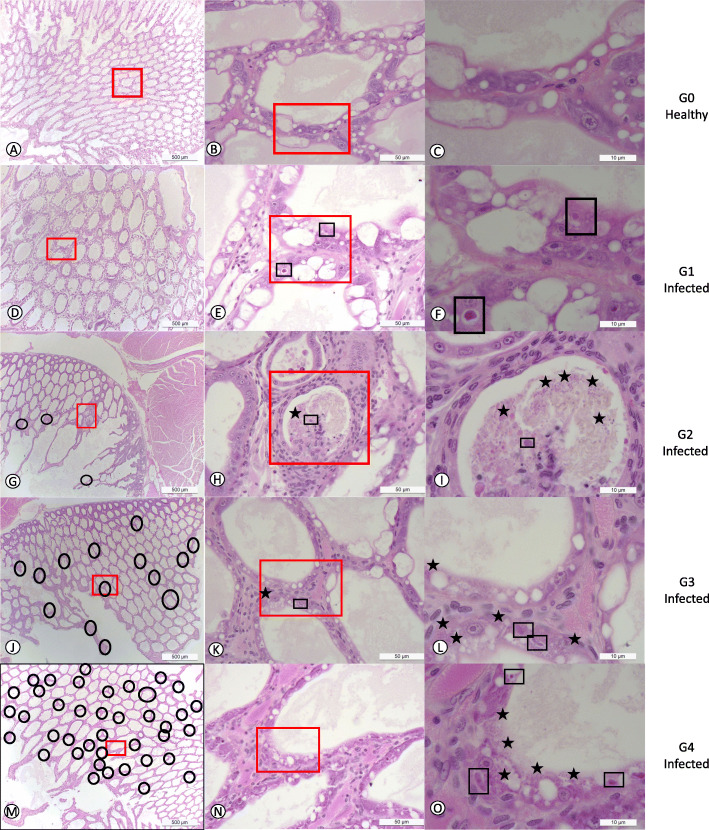
H&E (Mayer–Bennet hematoxylin and eosin-phloxine) staining of hepatopancreas tissue of *Penaeus vannamei* showing the presence of different stages of EHP infection **a**-**c**: grade 0; **d**-**f**: grade 1; **g**-**i**: grade 2; **j-l**: grade 3;. **m-o**: grade 4 of EHP infection. Mature spores are indicated by blue stars. Black square shows the typical regular-irregular plasmodium stage. Black circles show areas where EHP is present. Red square outlines regions that are magnified from the lower magnification. Scale bars are located in the inferior right part of each figure

The prevalence of EHP at 2 ppt, 15 ppt, and 30 ppt salinities in challenge #1 was 25, 33.3, and 25%, respectively. In challenge #2, the prevalence of EHP at 2 ppt, 15 ppt, and 30 ppt was 33.3, 30.0, and 87.5%, respectively. The degree of severity was higher at a salinity of 30 ppt in the second experiment (Table 2). In challenge #2, 50% of the EHP-infected population at 30 ppt displayed grade G3 (moderate to severe) and G4 (severe) lesions caused by the EHP infection. A strong association between salinities and *E. hepatopenaei*-infected shrimp was found with an Odds Ratio (OR) of 4.3 (*p* = 0.037). The prevalence of EHP in shrimp exposed to high salinity (30 ppt) was higher than shrimp exposed to low salinities (2 ppt and 15 ppt combined). The different grades of severity in this study are shown in Fig. 2. The Fig. 2 panels A, B, and C show tissue sections of HP at a low, medium, and high magnification of healthy shrimp from the control tank without showing any histological lesions of EHP or any other known pathogens. In contrast, panels D, E, and F show HP tissue sections displaying grade G1 of an EHP infection. A focalized region within the HP was observed (Fig. 2d). The affected tubule shows the distinctive cytoplasmic inclusion bodies in the cytoplasm of the affected epithelial tubule cells that correspond to the uninucleate meront stage (Fig. 2e -f). Figure 2 panels G, H, and I show grade G2 of an EHP infection (low to moderate). The focal presence of an EHP infection in few affected HP tubules epithelial cells were observed. Both, the meront stage and spores liberated into the lumen were observed (Fig. 2i). Figure 2 panels J, K, and L show a typical grade G3 of an EHP infection. Multifocal lesions in HP tubules epithelial cells were observed (Fig. 2j). In the affected tubules, the presence of both irregular multinucleated plasmodium and spores within the cytoplasm of the cuticular epithelial cells were observed (Fig. 2l). Figure 2 panels M, N, and O show the grade G4 of an EHP infection with multifocal tubules containing infected HP cells (Fig. 2m). Both multinucleated plasmodium and spores within the cytoplasm of the affected cells as well as spores within the lumina of the tubules were observed (Fig. 2o).

**Table 2:** A summary of EHP histological findings of *P. vannamei* exposed to EHP-containing fecal strings in two independent challenges at three different salinities. G0-G4 indicates the degree of severity (number of positive / number of total shrimp; [prevalence %](Lightner, 1996)

Challenge	Salinity			Combined data
	2 ppt	15 ppt	30 ppt	
#1	G4 (1/4) G0 (3/4) [25%]	G1 (2/6) G0 (4/6) [33.3%]	G1 (1/4) G0 (3/4) [25.0%]	G4 (1/14) G1 (3/14) G0 (10/14) [28.5%]
#2	G3 (1/6) G1 (1/6) G0 (4/6) [33.3%]	G1 (3/10) G0 (7/10) [30.0%]	G4 (3/8) G3 (1/8) G2 (2/8) G1 (1/8) G0 (1/8) [87.5%]	G4 (3/24) G3 (2/24) G2 (2/24) G1 (5/24) G0 (12/24) [50.0%]
Combined data	G4 (1/10) G3 (1/10) G2 (0/10) G1 (1/10) G0 (7/10) [30.0]	G4 (0/16) G3 (0/16) G2 (0/16) G1 (5/16) G0 (11/16) [31.2%]	G4 (3/12) G3 (1/12) G2 (2/12) G1 (2/12) G0 (4/12) [66.65]

### Detection of EHP in hepatopancreas by PCR

The hepatopancreas of SPF shrimp challenged with fecal strings obtained from EHP-infected shrimp had positive results for EHP using nested PCR in all of the tanks at the three different salinities. This confirms the presence of EHP in the treatment tanks for challenge #1 and challenge #2. In all three (3) salinities (2 ppt, 15 ppt, and 30 ppt) EHP was detected. The hepatopancreas tissue collected from the negative control treatment animals reared at the 2 ppt, 15 ppt, and 30 ppt salinities tested negative for EHP using nested PCR.

## Discussion

Since 2009, HPM caused by EHP has emerged as an economically important disease being highly prevalent in some of the major shrimp producing countries in SE Asia and, more recently in the western hemisphere in Venezuela [7]. In SE-Asian countries, along with white spot disease (WSD) and acute hepatopancreatic necrosis (AHPND), HPM remains a major threat contributing to significant economic losses in grow-out ponds. *Penaeus vannamei* is an euryhaline species which is raised in a wide range of conditions including high salinities (30 ppt), estuarine environments (10–20 ppt), and low salinities (2 ppt) [13]. In this study, we investigated the prevalence and severity of EHP in three salinities, high (30 ppt), medium (15 ppt), and low (2 ppt), under laboratory challenged conditions. The fecal strings, used as a source of EHP inocula, in the experimental challenges were sufficient to cause disease in shrimp maintained at different salinities as confirmed by histopathology and PCR. The results from this study provide a new EHP infection method through fecal strings via the fecal-oral route. Previous studies have shown some routes of infection included cohabitation [14, 15], reverse gavage, and direct hepatopancreas injection with EHP-inoculum [15]. In this study, we reported for the first time that feces can be an infectious contamination route. Due to the detritivorous behavior and the presence of undigested feed within the feces that could be around 25–30% [16], shrimp in treatment tanks were eating this source of food along with the EHP-infected spores mimicking the horizontal transmission observed at a farm level. EHP lesions, including the presence of plasmodium in the cytoplasm of infected cells and the mature spores within the cytoplasm or released spores in the lumen, were found by histopathology in EHP-challenged shrimp reared at different salinities. This unequivocally confirms EHP is able to cause an infection at a wide range of salinities, varying as much as 2 ppt to 30 ppt. When the initial inoculum used for the experimental challenge was low (i.e. 1 × 10^3^ copies of EHP/ ng of total fecal DNA), the HPM prevalence was similar (i.e. 25%) irrespective of the salinities (See Table 1). However, the prevalence of the EHP infection increased at 30 ppt salinity (87.5% prevalence), compared to 15 ppt (30.0% prevalence) and 2 ppt salinity (33.3% prevalence), when the inoculum level was increased from 1 × 10^3^ to 1 × 10^6^ copies of EHP/ ng of total fecal DNA in the challenge experiment (i.e. challenge #2). Dose-dependent challenge has been well documented for other shrimp pathogens such as AHPND and *Hepatobacter penaei* [9, 17]. In AHPND challenge tests, an infectious dose of 2.0 × 10^6^ CFU/ml is required to provoke high mortality (> 90%) in immersion challenge methods. In contrast, a lower dose (2.0 × 10^4^ CFU/ml) does not cause mortality nor histological lesions in the challenged population [9]. In the present study, the inoculum with low copy number (1.6 × 10^3^ copies of EHP/ ng of total fecal DNA) used in challenge #1 caused mild infections in the challenged shrimp. However, severe infections (Grades G3 to G4) and a higher prevalence occurred in the challenge #2 when the EHP copy number in the inoculum was higher (1.1 × 10^6^ copies of EHP/ ng of total fecal DNA).

The histological lesions in shrimp maintained at 30 ppt salinity were more severe. A moderate- severe grade of infection (G3-G4) was found in 50% of the shrimp affected. In contrast, only 16% of shrimp reared at salinity of 2 ppt showed grade G3 level of infection and 0% of shrimp reared at salinity of 15 ppt showed grade G2-G4 level of infection, according to the Lightner’s scale [18]. The difference in the severity of the EHP infection at the three different salinities was probably due to the differential effect of salinity on spore germination. One of the critical phases in the spore germination is the increase of intra-spore osmotic pressure. The difference in salinities led to a hypotonic environment at 2 ppt and 15 ppt compared to hypertonic environment at 30 ppt. It is possible that the hypertonic solution enhances the germination of the spore by increasing the spore activation process. He et al. [19] found a difference of eversion of the polar tube in different osmotic environments in *Encephalitozoon intestinalis*, an obligate intracellular microsporidium that causes gastrointestinal diseases in immunocompromised and immunocompetent people. Differences in polar tube germination associated with changes in salinity have also been reported by other researchers. De Graff et al. showed an increase in germination of *Nosema apis* spores at 0.5 N NaCl concentration [20]. Similarly, an increase of germination of *Nosema algerae* spores at 0.1 M NaCl concentration vs. 0.05 M NaCl was reported by other researchers [21]. In our study, the NaCl concentration was about 0.5 M, 0.2 M, and 0.03 M NaCl at 30 ppt, 15 ppt, and 2 ppt, respectively. The difference in NaCl concentrations perhaps could explain, in part, an effect on the germination of EHP spores and resulting prevalence levels.

Hardness is another variable that was different in the three salinities used in this study and could have been a factor that affected the spore germination. The hardness in low salinity (2 ppt) was about 240 mg/L (City of Tucson https://www.tucsonaz.gov/water/water-quality-reports-and-publications), vs. the marine water artificially prepared at 15 ppt and 30 ppt that was around 787 and 1575 mg/L respectively (Crystal Sea, Marinex). It has been reported that calcium is an important second messenger that activates many cell events and calcium influx might be, in part, responsible for the activation of microsporidian spore discharge at higher salinities [21].

In grow-out ponds of some EHP endemic areas in Asia, the salinity conditions are found to vary widely. For example, in India there are some shrimp farming areas in high and low salinity, and the prevalence of EHP seems to be lower at lower salinities (below 5 ppt), as observed during a shrimp disease survey in Andhra Pradesh in 2019 (Aranguren et al., Unpublished data). Similar conditions were recorded in two major shrimp farming areas in Venezuela, i.e. Maracaibo lake where the salinity is around 4–6 ppt and in Falcon state where the salinity varies from 36 to 40 ppt. In Venezuela, shrimp farming is not fully integrated, and the movement of nauplii and post-larvae between Falcon and Maracaibo lake area is a common practice. This suggests that EHP-infected PL and / or broodstock have been moved between these two zones. However, EHP has only been detected in the Falcon area where the salinities are high. In the Maracaibo’s lake where the salinities are low, EHP has not been reported. One possibility that has limited the spread of EHP could be the difference in water salinity.

## Conclusion

This study demonstrated that fecal strings from known EHP-infected shrimp could be used as a reliable source of inoculum to conduct EHP experimental infections via the fecal-oral route. An EHP infection can occur at a low salinity (i.e. 2 ppt) although the prevalence and the severity of infection is higher at a salinity of 30 ppt. These findings have implications in disease management in EHP-endemic areas.

## Methods

### Shrimp

Specific Pathogen Free (SPF) *Penaeus vannamei* were obtained from a commercial vendor in Florida, US. The SPF population has been screened for the last 2 years at the UA-APL without presence of any of the OIE-listed and non-listed diseases including EHP. The bioassays were carried out in the Aquaculture Pathology Laboratory (UA-APL) of The University of Arizona. The American Veterinary Medical Association (AVMA) guidelines were followed while processing shrimp for histopathology analysis and for euthanasia by salt water/ice slurry at the end of the experiment (https://www.avma.org/sites/default/files/2020-01/2020-Euthanasia-Final-1-17-20.pdf).

### Enterocytozoon hepatopenaei bioassay

The EHP isolate used in this study was obtained from a *P. vannamei* population originating in Thailand. Two independent EHP challenges were conducted. In both challenges, shrimp were maintained at three different salinities of 2 ppt, 15 ppt, and 30 ppt. For each experimental challenge, six 90-L tanks were filled with artificial seawater (Crystal Sea Marinex, Baltimore, Maryland) that corresponded to the three salinity levels with two replicates for each salinity treatment. Temperature was adjusted at 25 °C (±0.6) with measurements each morning with a NIST thermometer. pH was measured with a pH indicator strips (Baxter®) once a week with a range of 7.5–8.0. Salinity was adjusted by changing 3 parts of the salinity every hour from 25 ppt (initial salinity of the SPF *P. vannamei* population) down to 5 ppt. In order to achieve a salinity of 2 ppt from 5 ppt, the salinity part was changed every 2 h. Once set up, salinity was measured with a salt refractometer (Sper Scientific®) once a week over the length of the challenges. Ten (10) SPF *P. vannamei* (weights: 2.0–2.1 g) were stocked in each tank for the experimental infection, respectively. Three 90-L control tanks were set up for each salinity as negative control treatment.

### Inoculum preparation

Over the course of the two challenges, the EHP-infected shrimp were fed daily at 5% of the total biomass. Fecal strings were collected daily by siphoning the fecal strings from the EHP-infected tanks into a tube 1 h after feeding. In order to prepare the inoculum for challenge #1, the EHP-infected fecal strings were pooled from three tanks with 5 shrimp per tank (mean weight 12.5 g). For challenge #2, EHP inoculum was prepared by pooling fecal strings from one 1000 L tank containing 60 EHP-infected shrimp (mean weight 9.0 g). The salinity of the EHP-infected tanks used as inoculum sources were constant for both challenges at 25 ppt (±1.0). After siphoning the fecal strings from the tanks, it was weighed and aliquoted in seven equal parts. Six of the aliquots were used as the inoculum for the experimental infection of each treatment tank. Each of the six aliquots were added to the treatment tanks by mixing the tube contents into the water of the treatment tanks. The remaining aliquot was preserved in 95% ethanol for EHP detection by PCR following a previously published protocol [4].

Starting 1 h after the inoculation process, shrimp were fed once a day at 2% of the total biomass with a commercial pelleted feed (Rangen 35%, Buhl, Idaho). This process forced the shrimp to eat first the inocula that contained EHP-infectious fecal strings. Survival was recorded daily from the start of the challenge. At the end of the challenge, shrimp samples from the challenge #1 and #2 from each treatment tank were fixed in Davidson’s (AFA) fixative [22]. Details of the number of samples for H&E ranged from 4 to 6 in the challenge #1 and from 6 to 10 in the challenge #2. The details of the number of samples is described in Table 2. Pooled hepatopancreas tissue from 3 to 4 shrimp were collected from each tank in challenge # 1 and #2 for a total of 6 pools. Samples were preserved in 95% ethanol for EHP detection by PCR. The duration of the challenges was 20 days and 26 days for the challenge #1 and #2, respectively.

### Histopathology

The Davidson’s alcohol-formalin-acetic acid (AFA)-fixed samples were processed, embedded in paraffin, and sectioned (5 μm thick) in accordance with standard methods [18, 22]. After staining with hematoxylin and eosin (H&E), the sections were analyzed by light microscopy. Severity grades of the EHP infection/lesion ranged from G0-G4 according to Lightner (1996) with G0 being absence of the disease and G4 being presence of severe lesions and advanced tissue destruction.

### Conventional and quantitative PCR in detecting and quantifying EHP

Two different PCR methods were used in this study: Conventional nested PCR for EHP detection and real-time PCR for quantitation of EHP load. EHP detection was carried out by a conventional nested PCR that targets the Spore Wall Protein 1 (SWP1) gene. The primers for the first step are: SWP 1F/1R (1F: 5′-TTG CAG AGT GTT GTT AAG GGT TT-3, 1R: 5′-CAC GAT GTG TCT TTG CAA TTT TC-3′). The primers generate a 514 bp amplicon. The primers for the nested step are: SWP 2F/2R (2F:5′-TTG GCG GCA CAA TTC TCA AAC A-3′, 2R:5′-GCT GTT TGT CTC CAA CTG TAT TTG A 3′) which generates a 148 bp amplicon. The PCR protocol for the first PCR reaction consisted of a 5-min initial denaturation at 95 °C followed by 30 cycles of denaturation for 30 s at 95 °C, annealing for 30 s at 58 °C and extension for 45 s at 68 °C with a final 5-min extension step at 68 °C. For the second (nested) PCR step, the template consisted of 1 μl of the final reaction solution from the first PCR step. The PCR protocol for the second, nested PCR reaction consisted of an initial denaturation at 95 °C for 5 min followed by 20 cycles of 30 s denaturation at 95 °C, 30 s annealing at 64 °C and 20 s extension at 68 °C with a final extension for 5-min at 68 °C [23]. For EHP quantification by real-time PCR, primers F:157 (5′-AGT AAA CTA TGC CGA CAA-3′) and R:157 (5′-T TAA GCA GCA CAA TCC-3′), and a TaqMan probe (5-FAM-TCC TGG TAG TGT CCT TCC GT-TAMRA-3′) were used. The real-time PCR protocol consisted of a 20 s initial denaturation at 95 °C followed by 40 cycles of denaturation for 1 s at 95 °C and annealing/extension for 20 s at 60 °C, following a previously published method with minor modifications [24].

### Statistical analysis

The EHP copy number obtained from the quantification analysis was transformed to log base 10 value prior to carrying out statistical analysis using SPSS v16.0. A one-way ANOVA with Tukey’s multiple comparison were performed and *P*-value < 0.05 was considered as statistically significant. The association between salinities and *E. hepatopenaei* was determined by calculating the odds ratio (OR) and the statistical significance at α = 0.05 (*n* = 38) (Dean et al. 2013).

## Data Availability

The datasets analyzed during the current study are available from the corresponding author on reasonable request.

## Abbreviations

SE Asia: South East Asia
SWP: Spore Wall Protein
AFA: Alcohol-Formalin-Acetic acid
SPF: Specific Pathogen Free
ppt: part per thousand
